# Disability and Participation in Colorectal Cancer Screening: A Systematic Review and Meta-Analysis

**DOI:** 10.3390/curroncol31110517

**Published:** 2024-11-10

**Authors:** Giovanni Emanuele Ricciardi, Rita Cuciniello, Emanuele De Ponti, Carlo Lunetti, Flavia Pennisi, Carlo Signorelli, Cristina Renzi

**Affiliations:** 1 PhD National Programme in One Health Approaches to Infectious Diseases and Life Science Research, Department of Public Health, Experimental and Forensic Medicine, University of Pavia, 27100 Pavia, Italy; 2 School of Medicine, Università Vita-Salute San Raffaele, 20132 Milano, Italy; 3 Research Department of Behavioural Science and Health, University College London, London WC1E 6BT, UK

**Keywords:** disability, colorectal cancer, screening

## Abstract

Background: The aim of this study is to assess the impact of disability on participation in CRC screening and to determine the overall effect size. Methods: We conducted a systematic review and meta-analysis to compare CRC screening participation in individuals with and without disabilities. The search encompassed five databases (PubMed, EMBASE, Scopus, Google Scholar, medRxiv). Pooled estimates were calculated for each type of CRC screening and disability categories to synthesize the findings. The participation in CRC screening was derived using a random effects model. Results: A total of 20 articles were included, most of them from the USA. Based on pooled estimates, individuals with disabilities have lower odds of undergoing CRC screening versus those without disabilities (OR = 0.80, 95%CI 0.73–0.87). Analysis by screening type indicated that individuals with a disability have lower odds of a fecal occult blood test or a fecal immunochemical test (OR: 0.72, 95%CI 0.65–0.81), with no significant difference for a colonoscopy. Individuals with intellectual disabilities had significantly lower rates of CRC screening participation (OR = 0.65, 95%CI 0.53–0.79), especially for FOBT/FIT (OR = 0.58, 95%CI 0.49–0.69). Conclusions: Disparities exist for CRC screening participation in people with disabilities. Further research and coordinated efforts are essential to develop interventions for improving early cancer diagnosis for this non-negligible patient group.

## 1. Introduction

Colorectal cancer (CRC) ranks as the third most prevalent cancer and the second leading cause of cancer-related deaths worldwide. In 2022, it was estimated that over 1.9 million new cases of CRC were diagnosed, with more than 900,000 deaths attributed to the disease globally [1]. CRC screening programs, such as colonoscopy, fecal occult blood testing (FOBT), and fecal immunochemical testing (FIT), are vital for the early detection of colorectal cancer. When combined with prompt diagnosis and treatment, these screenings can substantially lower mortality rates. Participation in CRC screening programs is associated with lower CRC-specific mortality by up to 73%, with the 10-year colonoscopy being the most effective in reducing mortality [2].

Despite the implementation of population-wide screening programs, in many countries, significant disparities in screening uptake persist. Several factors contribute to lower screening participation, including socioeconomic status, access to healthcare, and patient knowledge and beliefs about CRC [3,4]. Research also identified an association between disability and participation in cancer screening [5,6,7], with mixed findings and variations by disability type [8,9]. A disability is any health condition, including physical, sensorial, and mental health conditions, interfering significantly with daily activities [10]. The WHO estimated 1.3 billion people live with a disability, representing 16% of the world’s population [11].

People with a disability can encounter greater barriers when accessing healthcare services, including difficulties accessing cancer preventive services and medical care when needed [12,13]. These encompass physical, emotional, cognitive, sensorial, or communication barriers, as well as economic challenges, which can vary by type of disability.

A systematic review showed that women with disabilities have lower odds of attending breast and cervical cancer screening compared to women without disabilities [14]. To our knowledge, there is no systematic review of the association between disability and CRC screening. The aim of this study is to systematically review the impact of disabilities on participation in CRC screening and to determine the overall effect size through a meta-analysis.

## 2. Materials and Methods

### 2.1. Protocol

This systematic literature review examined CRC screening participation among people with and without disabilities. The study protocol was registered with the International Prospective Register of Systematic Reviews (PROSPERO), CRD42023468480, on 13 October 2023. The review was conducted and reported in accordance with the Cochrane Handbook for Systematic Reviews guidelines [15] and the Preferred Reporting Items for Systematic Reviews and Meta-Analyses (PRISMA) statement [16]. The PRISMA checklist is available in the Supplementary Materials (Table S1) [17].

### 2.2. Definition of Disability

Our search focused on disability, as defined by the World Health Organization International Classification of Functioning, Disability, and Health (WHO-ICF) framework [18]. Disability will encompass physical, sensory, cognitive, and psychosocial impairments associated with activity limitations or participation restrictions.

### 2.3. Search Strategy

A systematic search was performed across five databases (MEDLINE/PubMed, EMBASE, Scopus, Google Scholar, and medRxiv). Search engines were used to find published evidence without time restrictions. Our search included specific diagnostic codes for conditions and impairments considered likely to be disabling, such as “psychosis”, “visual impairment”, and “functional hearing loss”. In addition to searching databases, further pertinent studies were identified by examining the reference lists of included articles. The final search update was conducted on 23 May 2024. The search strategy for MEDLINE/PubMed is reported in the Supplementary Materials (Table S2).

Reports were eligible for inclusion if they quantitatively assessed the participation of CRC screening (colonoscopy, FOBT, and FIT) in persons with and without disabilities falling within the age range eligible for screening. All inclusion and exclusion criteria are described in Table 1.

We included quantitative observational or interventional studies, with no geographical or publication date restrictions. We excluded studies if they did not compare screening participation between individuals with disabilities and those without disabilities. Additionally, we excluded qualitative studies, review studies, and studies lacking clarity in reporting effect measures, such as missing information on lower or upper limits or the ability to calculate these values. Non-English language articles were also ineligible for inclusion.

### 2.4. Data Collection and Synthesis of the Results

The results from the searches were transferred to Zotero [19]. Duplicates were removed automatically using Zotero and double checked manually by one author (GER). Subsequently, articles were exported as a Research Information Systems file (.ris) and imported into Rayyan [20] for title and abstract screening. Abstracts and full-text articles were independently screened by four reviewers (GER, RC, EDP, and CL) for eligibility. Any disagreements were resolved through discussion or adjudication by a third reviewer if required.

Data extraction was undertaken using a data extraction template by 3 authors (GER, EDP, and RC) using Microsoft Excel. We extracted key characteristics from each included study (authors, year of study, country, study design, and dimension), information on the disability (type and definition), and which screening was used (FOBT/FIT, colonoscopy/sigmoidoscopy, both). To further enhance the clarity of interpretation, as well as to facilitate a more systematic analysis of the results, disabilities were classified under 7 categories, as follows:

- Functional disability is defined as having limitations or impairments in performing activities of daily living due to various health conditions not included in the other categories, such as heart disease (e.g., heart failure), respiratory conditions (e.g., chronic obstructive pulmonary disease), liver disease (e.g., cirrhosis), or kidney disease (e.g., chronic kidney failure);
- Physical disability is defined as a limitation on a person’s physical functioning, mobility, dexterity, or stamina;
- Hearing impairment is defined as a deviation or change for the worse in either auditory structure or auditory function, usually outside the range of normal;
- Visual impairment is defined as the partial or total inability of visual perception;
- Intellectual disability is defined as significant limitations in both intellectual functioning (problem solving and judgment) and adaptive behavior (communication skills and social participation);
- Learning disability is defined as disorders that affect the ability to understand or use spoken or written language, perform mathematical calculations, or direct attention;
- Psychosocial disability is defined as a psychiatric or mental health diagnosis, a history of psychiatric prescription, or self-reported mental status.

### 2.5. Risk of Bias

Quality assessment was independently assessed by four raters (GER, RC, EDP, CL) applying the Critical Appraisal Checklist for prevalence studies proposed by The Joanna Briggs Institute (JBI) [21]. This checklist served as a comprehensive evaluation tool for examining the methodological rigor of each study. Studies were assigned a risk of bias rating based on their adherence to the checklist criteria and were categorized as a low or high risk of bias.

### 2.6. Data Synthesis and Meta-Analysis

To synthesize the findings across studies, pooled estimates were calculated for each type of CRC screening and disability categories. The participation of CRC screening among individuals with and without disabilities was derived using a random effects meta-analysis model. This approach accounts for potential between-study heterogeneity, which can arise from factors such as variations in study design, sampling methods, disability assessment tools, and outcome measures. The I^2^ and τ^2^ statistics were employed to assess the extent of heterogeneity among the included studies. All statistical analyses were conducted using Stata version 18 and package meta [22].

## 3. Results

### 3.1. Literature Search

**Figure 1:**
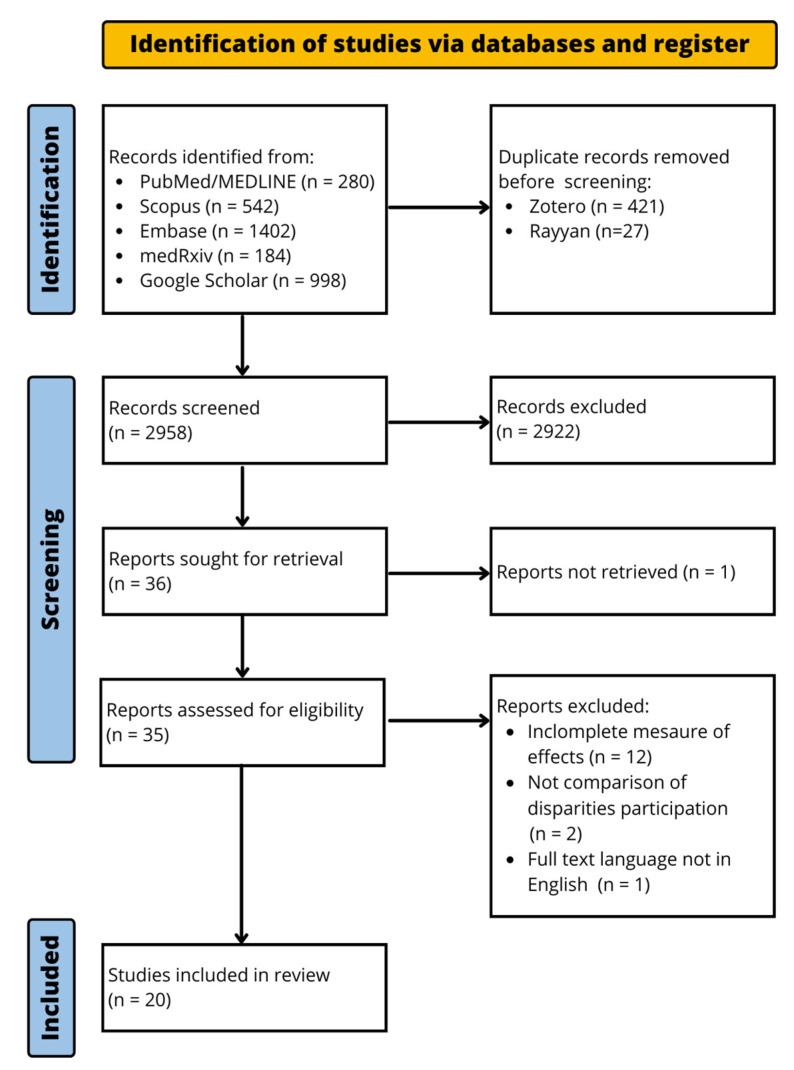
PRISMA flow diagram.

A total of 3406 records were retrieved from five electronic databases, with no additional records identified through backward citation searches. After removing duplicates and screening titles and abstracts for relevance, 36 records were selected for full-text review. After a thorough evaluation of the records, sixteen records were excluded: one due to unavailability of the full text, twelve for incomplete reporting of impact measures, two for lack of comparative data on participation disparities, and one due to being non-English. A total of 20 studies met the inclusion criteria, focusing on CRC screening participation among individuals with and without disabilities. Figure 1 shows the study selection flow diagram.

### 3.2. Study Characteristics

Table 2 reports the characteristics of the included studies. Of the twenty included studies, thirteen were conducted in the USA [23,24,25,26,27,28,29,30,31,32,33,34,35] and two in South Korea [36,37]. The five remaining studies were conducted, respectively, in the UK [38], Norway [39], Canada [40], Taiwan [41], and Japan [42]. The majority of the studies had nationwide coverage [24,25,27,28,29,30,31,33,36,37,38,41,42], while the others focused on specific regions within countries [23,26,32,35,39,40]. Moreover, one study included only women [38]. A total of 55% of the studies were cross-sectional [24,25,27,28,29,32,33,34,37,40,42], and 35% of the studies were cohort studies [23,26,30,35,36,38,41]. The remaining studies were case–control [39] and mix method [31].

### 3.3. Type of Disability

In twelve studies, disability was defined based on ICD codes in the patient’s medical records [23,24,25,26,30,31,35,36,37,39,40,41], whereas in eight studies, disability was defined based on self-reported answers to standardized questionnaires [27,28,29,32,33,34,38,42]. Psychosocial disability, assessed as a psychiatric or mental health diagnosis, a history of psychiatric prescription, or self-reported mental status, was the most frequently examined disability and was reported in eight studies out of the twenty included [23,30,31,35,37,38,39,41]. Intellectual disability was reported in seven studies [26,33,34,37,38,40,41]. Functional disability, considered as having limitations or impairments in performing activities of daily living due to various health conditions, was recorded in six studies [25,27,37,38,41,42], as well as visual impairment [24,26,33,37,38,41]. The other categories reported were physical disability in five studies [26,29,33,37,41], hearing impairment in four studies [33,37,38,41], and learning disability in two studies [37,41]. Moreover, eight studies included two or more types of disabilities [26,28,32,33,36,37,38,41]. Demographic and socioeconomic characteristics provided by each study are reported in the Supplementary Materials (Table S3), while one study evaluated transport access [38], and no study evaluated institutionalization.

### 3.4. Type of CRC Screening

The type of CRC screening evaluated in the studies is shown in Table 3. Fourteen studies evaluated and reported adherence to any CRC screening [23,24,25,26,27,28,29,30,31,32,33,36,39,40], five studies only to FOBT/FIT [35,37,38,41,42], and one study only to colonoscopy/sigmoidoscopy [34]. The age range of patients included in the studies is also shown in Table 3 and was defined according to national guidelines for the years when each study was conducted.

### 3.5. Colorectal Cancer Screening Participation in Individuals with a Disability

Table 3 shows the number of individuals with and without a disability and screening participation in each study. Overall, the studies included a total of 59 million participants without a disability and about 5 million participants with a disability. Since some studies did not clarify whether patients with disabilities could be classified into multiple categories, the reported number of participants with disabilities is an excess estimate.

### 3.6. Risk of Bias Analysis

Seventeen studies were deemed to have a low risk of bias, and three studies had a high risk of bias (see Table S4) [31,35,42]. In most cases, studies that have a high risk of bias were due to the targeting of population subgroups (e.g., veterans or insured patients), which may not appropriately address the general population. In some studies, the sample size was not adequate, and socio-demographic variables were not homogeneously distributed (e.g., studies targeting veterans mainly included male participants).

### 3.7. Colorectal Cancer Screening Participation in People with Disabilities

**Figure 2:**
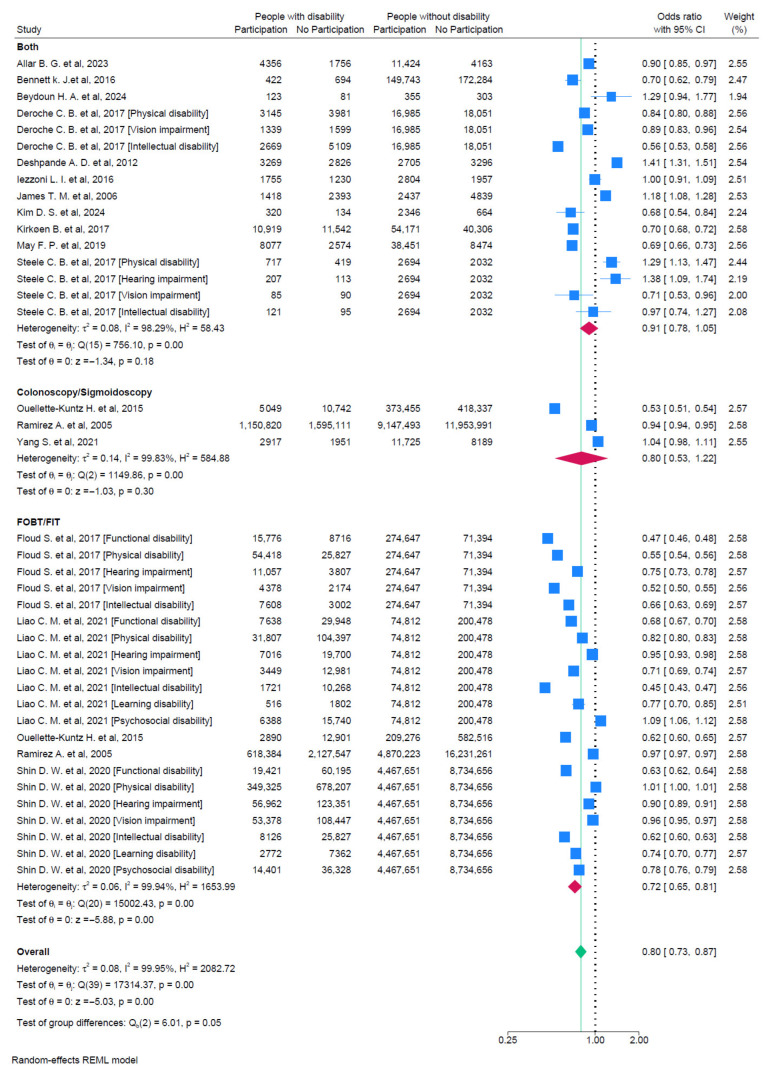
Pooled odds ratio estimates of any CRC screening participation by disability status [23,24,25,26,27,28,29,30,32,33,34,36,37,38,39,40,41]. Blue squares show the odds ratios (ORs) for individual studies, with horizontal lines for 95% confidence intervals. Red diamonds represent the pooled ORs for each screening method subgroup and the green diamond indicates the overall pooled OR for all studies. The green dashed line indicates the overall pooled OR, allowing comparison with subgroup and individual study ORs. The vertical dotted line indicates the null effect threshold.

A total of 40 data points from 17 studies were included in the pooled analysis of CRC screening participation (Figure 2). The combined estimate indicated that individuals with disabilities have lower odds of participation in CRC screening compared to those without disabilities (OR = 0.80, 95%CI 0.73–0.87). Individual estimates ranged from 0.45 to 1.38, and there was strong evidence for heterogeneity (I^2^ = 99.95%; τ^2^ = 0.08). The analysis by type of screening showed that people with a disability have lower odds in the case of a FOBT/FIT (OR = 0.72, 95%CI 0.65–0.81), but this is not so for colonoscopy or sigmoidoscopy (OR = 0.80, 95%CI 0.53–1.22).

Table 4 shows the subgroup analysis we made by disability type and CRC screening type. It was not possible to make subgroup analyses about the participation in the case of colonoscopy or sigmoidoscopy due to the insufficient number of available studies.

**Figure 3:**
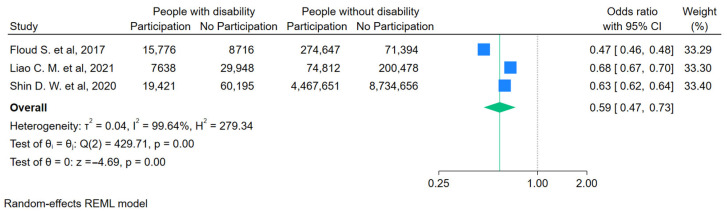
Pooled odds ratio estimates of FOBT or FIT participation by functional disability status [37,38,41]. Blue squares show the odds ratios (ORs) for individual studies, with horizontal lines for 95% confidence intervals and the green diamond indicates the overall pooled OR for all studies. The green dashed line indicates the overall pooled OR, allowing comparison with individual study ORs. The vertical dotted line indicates the null effect threshold.

**Figure 4:**
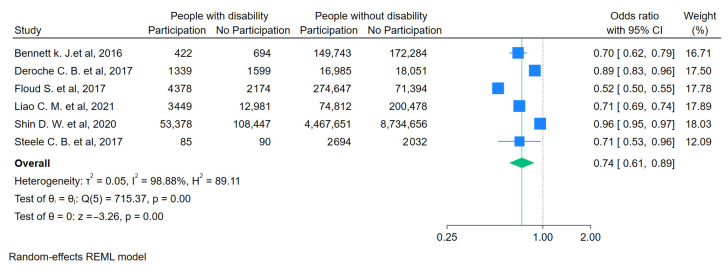
Pooled odds ratio estimates of any CRC screening participation by visual impairment status [24,26,33,37,38,41]. Blue squares show the odds ratios (ORs) for individual studies, with horizontal lines for 95% confidence intervals and the green diamond indicates the overall pooled OR for all studies. The green dashed line indicates the overall pooled OR, allowing comparison with individual study ORs. The vertical dotted line indicates the null effect threshold.

**Figure 5:**
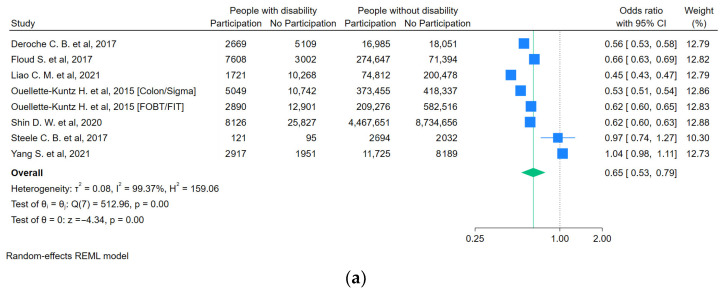

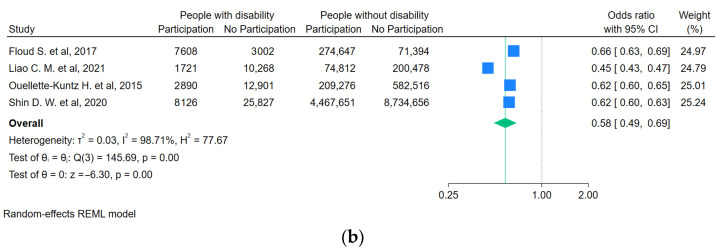
(**a**) Pooled odds ratio estimates of any CRC screening participation by intellectual disability status [26,33,34,37,38,40,41]. (**b**) Pooled odds ratio estimates of FOBT or FIT participation by intellectual disability status [37,38,40,41]. Blue squares show the odds ratios (ORs) for individual studies, with horizontal lines for 95% confidence intervals and the green diamond indicates the overall pooled OR for all studies. The green dashed line indicates the overall pooled OR, allowing comparison with individual study ORs. The vertical dotted line indicates the null effect threshold.

**Figure 6:**
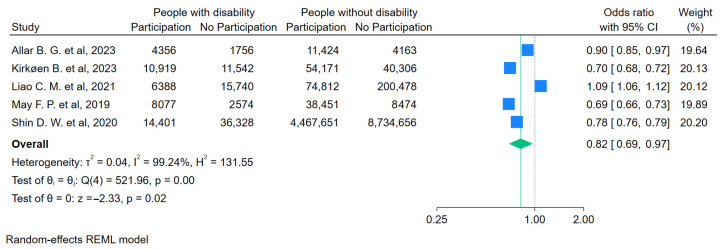
Pooled odds ratio estimates of any CRC screening participation by psychosocial disability status [23,30,37,39,41]. Blue squares show the odds ratios (ORs) for individual studies, with horizontal lines for 95% confidence intervals and the green diamond indicates the overall pooled OR for all studies. The green dashed line indicates the overall pooled OR, allowing comparison with individual study ORs. The vertical dotted line indicates the null effect threshold.

For people with a functional disability (Figure 3), the likelihood of getting screened for FOBT/FIT was lower compared to people without a disability (OR = 0.59, 95%CI 0.47–0.73). For people with visual impairments (Figure 4), the likelihood of getting any screening was lower (OR = 0.74, 95%CI 0.61–0.89). For people with an intellectual disability (Figure 5a,b), the likelihood of getting any screening was significantly lower (OR = 0.65, 95%CI 0.53–0.79) and even lower for FOBT/FIT (OR = 0.58, 95%CI 0.49–0.69). For people with a psychosocial disability (Figure 6), the likelihood of getting any screening was also lower (OR = 0.82, 95%CI 0.69–0.97). The additional analyses are accessible in the Supplementary Materials (Figures S1–S4).

## 4. Discussion

Overall, people with disabilities were 20% less likely to undergo CRC screening. Analyzing different disability types separately showed that people with an intellectual disability were less likely to undergo CRC screening. All studies included in this review were conducted in high-income countries, with the majority referring to the United States.

The results of this meta-analysis are consistent with what the literature offers on the subject. A mixed-methods systematic review suggests that individuals with disabilities had a significantly lower participation in cancer screening compared to those without disabilities [43]. However, this review included seventeen studies, but only one of them focused on CRC screening. Also, a systematic review and meta-analyses showed that women with disabilities are less likely to attend breast and cervical screenings compared to women without disabilities [14]. Therefore, our study confirms the disparities in screening services for people with disabilities. Qualitative research shows that people with a disability face barriers in adhering to recommended screening protocols. These barriers include inadequate knowledge of recommendations, language obstacles, logistical challenges, and cultural beliefs [44]. Financial constraints and apprehensions about bowel preparation for colonoscopy and sigmoidoscopy may also act as deterrents [45,46].

The barriers that people with a disability face may vary depending on the type of disability. For instance, current communication methods are unable to meet the needs of individuals with intellectual disabilities [26,40,47]. Some studies about psychosocial disabilities suggest a substantial and significant disparity in the level of secondary cancer prevention between the general population and people with disabilities, resulting in a reduction in life expectancy by several years [48,49,50]. In our review, we found that also people with a visual impairment have a significantly low likelihood of undergoing CRC screening. A prior study on barriers to healthcare access for people with visual impairment indicated increased issues with healthcare access due to cost, insurance coverage availability, transportation problems, and service refusal by providers [51]. However, there is some disagreement in the research regarding participation rates among people with disabilities. Some studies have reported higher participation rates [8,9,28]. One possible reason for these higher rates is that people with a disability often have more contact with primary care physicians to manage other health issues. However, other studies have found that patients with functional limitations or poor perceived health status are less likely to follow cancer screening recommendations [52,53,54,55,56,57,58,59].

We found that people with functional and intellectual disabilities have a lower likelihood of undergoing FOBT/FIT. One possible explanation for this difference could be that FOBT/FIT is a screening test that patients can administer themselves, whereas a colonoscopy is a procedure conducted in a hospital setting. For example, in the USA, individuals receive the kit for FOBT/FIT testing and send biological material through the mail service [60]. This could be an additional difficulty for individuals with disabilities in attending screening.

Our review study presents several strengths and limitations that should be considered. Firstly, we identified only three studies that investigated the likelihood of undergoing colonoscopy, so it was not possible to explore the differences between screening types by specific disability. Further research is required to examine colonoscopy adherence among individuals with disabilities. Secondly, we did not find any studies examining the likelihood of individuals with a disability in institutional settings participating in CRC screening programs. This highlights a significant gap in current research and suggests a potential area for a future investigation. Thirdly, the majority of the studies were conducted in the USA, making it difficult to generalize the evidence to other counties and health contexts. Additionally, some studies focus on particular population subgroups, such as veterans, insured patients, or urban residents, and their findings may not be universally applicable to the general population.

Another notable aspect is the high heterogeneity I^2^ observed, which is likely attributable to the different methods used to evaluate the disability. Most of the studies used clinical diagnostic criteria to assess visual impairment, hearing loss, and other psychiatric or mental health disorders. However, some studies relied on self-assessment of the disability without specifying the nature of the disability. The most common way to measure the disability has been to ask people to self-report their functioning, including body functions and activity components of the ICF, such as vision, hearing, and mobility. There are several validated tools for disability assessment, such as the Washington Group Short Set (WGSS) [60], the Washington Group Extended Set of Functioning (WGES) [61], the Model Disability Survey (MDS) [62], the Equality Act Disability Definition (EADD) [63], and the Global Activity Limitation Instrument (GALI) [64]. Each of these tools has a number of advantages and disadvantages. Additionally, the heterogeneity statistic I^2^ is influenced by the precision and size of the studies included, whereas the heterogeneity statistic τ^2^ is not affected by the number or size of the studies [65].

The review’s strengths lie in the comprehensive search strategy employed, which embraced a holistic approach to defining disability through the use of search terms aligned with the ICF framework. This strategy also encompassed clinical diagnoses and conditions relevant to disability. Moreover, we included five databases and gray literature for enhancing the robustness of the approach. Another significant strength lies in the large sample size, comprising approximately 59 million individuals without disabilities and about 5 million with disabilities, allowing us to stratify the data by disability type and screening approach. Adhering to the PRISMA guideline also provided this review with methodological rigor.

## 5. Future Directions

To improve adherence to CRC screening among individuals with various forms of disabilities, a comprehensive set of strategies should be implemented to address the specific barriers. First and foremost, healthcare providers could benefit from training to gain a deeper understanding of the specific needs of individuals with disabilities. This would enable them to appropriately communicate and inform these patients about preventive care and the critical importance of CRC screening.

In addition, improving the accessibility of screening is essential. This includes incorporating ramps, elevators, and mobility aids to facilitate access to healthcare services for those with physical impairments. For individuals who face logistical challenges, dedicated transportation services should be offered to help them reach screening centers. Alternatively, certain phases of the screening process, such as bowel preparation for a colonoscopy or support with the FOBT/FIT test, could be arranged at people’s homes or their GP practice.

Effective communication is also key. Informational materials should be provided in accessible formats such as braille, audio, or videos with subtitles and sign language for individuals with sensory disabilities. For those with cognitive disabilities, the use of simple, clear language is crucial to ensure understanding. Additionally, programs that actively engage caregivers are vital, equipping them with the necessary information and tools to assist individuals with disabilities in understanding the importance of CRC screening and facilitating their participation.

Targeted awareness campaigns are another critical component, specifically designed to address the challenges faced by people with disabilities. These campaigns, delivered also with the participation of people with a disability, should emphasize the importance of CRC screening and demonstrate how participation is possible, even in the face of potential obstacles. For individuals who may experience anxiety or fear surrounding the screening process, offering psychological and emotional support can make the experience less stressful and more manageable. Personalized recall programs should also be developed, taking into account the unique needs of people with disabilities. These programs would feature regular reminders and flexible booking options, making the screening process more accessible and less burdensome. Finally, collaboration with disability organizations and associations is essential in co-designing tailored interventions and strategies that effectively address the specific needs of this population.

## 6. Conclusions

The review highlights significant disparities in the participation of CRC screening in individuals with disabilities compared to the general population. Urgent public health actions are needed to ensure equitable access to preventive and early diagnosis services. Different stakeholders, including policymakers, primary care, specialists, and prevention services, must address these inequalities by incorporating disability-inclusive strategies within the healthcare system and routine clinical practice. More qualitative studies are essential to better define the specific barriers faced by people with disabilities and to develop interventions tailored to each type of disability.

## Figures and Tables

**Table 1:** A priori defined inclusion and exclusion criteria according to the PICOS framework.

Search Strategy	Details
Inclusion criteria	P: People with a disability who fall within the age range eligible for CRC screening (≥50 years) I: Participation in CRC screening C: Individuals without disabilities who meet the criteria for CRC screening programs O: Cancer screening participation S: Original quantitative epidemiological studies (experimental and observational)
Exclusion criteria	Studies that fail to compare disparities in screening participation between individuals with disabilities and those without disabilities. Review studies and studies lacking clarity in reporting effect measures, such as missing information on lower or upper limits or the ability to calculate these values
Language filter	English
Time filter	Without time restrictions
Databases	MEDLINE-PubMed, EMBASE, Scopus, Google Scholar, and medRxiv

**Table 2:** Study characteristics.

Author	Study Location	Dimension Study	Study Design	Definition of Disability	Type of Disability *							
					A	F	P	H	V	I	L	Ps
Allar B. G. et al. (2023) [23]	USA	Regional	Cohort study	Medical record								x
Bennett K. J. et al. (2016) [24]	USA	National	Cross-sectional study	Medical record					x			
Beydoun H. A. et al. (2024) [25]	USA	National	Cross-sectional study	Medical record		x						
Deroche C. B. et al. (2017) [26]	USA	Regional	Cohort study	Medical record			x		x	x		
Deshpande A.D. et al. (2012) [27]	USA	National	Cross-sectional study	Self-reported questionnaire		x						
Floud S. et al. (2017) [38]	UK	National	Cohort study	Self-reported questionnaire		x	x	x	x	x		
Iezzoni L. I. et al. (2016) [28]	USA	National	Cross-sectional study	Self-reported questionnaire	x							
James T. M. et al. (2006) [29]	USA	National	Cross-sectional study	Self-reported questionnaire			x					
Kim D. S. et al. (2024) [36]	South Korea	National	Cohort study	Medical record	x							
Kirkøen B. et al. (2023) [39]	Norway	Regional	Case-control study	Medical record								x
Liao C. M. et al. (2021) [41]	Taiwan	National	Cohort study	Medical record		x	x	x	x	x	x	x
May F. P. et al. (2019) [30]	USA	National	Cohort study	Medical record								x
Murphy K. A. et al. (2021) [31]	USA	National	Mix-method study	Medical record								x
Ouellette-Kuntz H. et al. (2015) [40]	Canada	Regional	Cross-sectional study	Medical record						x		
Ramirez A. et al. (2005) [32]	USA	Regional	Cross-sectional study	Self-reported questionnaire	x							
Saito T. et al. (2024) [42]	Japan	National	Cross-sectional study	Self-reported questionnaire		x						
Shin D. W. et al. (2020) [37]	South Korea	National	Cross-sectional study	Medical record		x	x	x	x	x	x	x
Steele C. B. et al. (2017) [33]	USA	National	Cross-sectional study	Self-reported questionnaire			x	x	x	x		
Yang S. et al. (2021) [34]	USA	National	Cross-sectional study	Self-reported questionnaire						x		
Yarborough B. J. H. et al. (2018) [35]	USA	Regional	Cohort study	Medical record								x

* A = any disability; F = functional disability; P = physical disability; H = hearing impairment; V = visual impairment; I = intellectual disability; L = learning disability; Ps = psychosocial disability.

**Table 3:** Estimate of the association between the presence of any disability and CRC screening.

Authors	Type of Screening	Age Range (Years)	Participants		Participation (%)	
			With Disability	Without Disability	With Disability	Without Disability
Allar B. G. et al. (2023) [23]	Any	50–75	[Psychosocial] = 6112	15,587	71.3	73.3
Bennett K. J. et al. (2016) [24]	Any	50–75	[Visual impairment] = 1116	322,027	37.8	46.5
Beydoun H. A. et al. (2024) [25]	Any	≥50	[Functional] = 204	658	60.1	53.9
Deroche C. B. et al. (2017) [26]	Any	50–75	[Physical] = 7126 [Visual impairment] = 2938 [Intellectual] = 7778	35,036	44.1 45.6 34.3	48.5
Deshpande A.D. et al. (2012) [27]	Any	≥50	[Functional] = 6905	6001	53.6	45.1
Floud S. et al. (2017) [38]	FOBT/FIT	60–74	[Functional] = 24,492 [Physical] = 80,245 [Hearing impairment] = 14,864 [Visual impairment] = 6552 [Intellectual] = 10,610	346,041	64.4 67.8 74.4 66.8 71.7	79.4
Iezzoni L. I. et al. (2016) [28]	Any	50–75	[Any disability] = 2985	4761	58.8	58.9
James T. M. et al. (2006)[29]	Any	≥50	[Physical] = 3811	7276	37.2	33.5
Kim D. S. et al. (2024) [36]	Any	50–79	[Any disability] = 454	3010	70.5	77.9
Kirkøen B. et al. (2023) [39]	Any	≥50	[Psychosocial] = 22,461	94,477	48.6	57.3
Liao C. M. et al. (2021) [41]	FOBT/FIT	50–69	[Functional] = 37,586 [Physical] = 136,204 [Hearing impairment] = 26,716 [Visual impairment] = 16,430 [Intellectual] = 11,989 [Learning] = 2318 [Psychosocial] = 22,128	275,290	20.3 23.4 26.3 21.0 14.4 22.3 28.9	27.2
May F. P. et al. (2019) [30]	Any	50–75	[Psychosocial] = 10,651	46,925	75.8	81.9
Murphy K. A. et al. (2021) [31]	Any	50–64	[Psychosocial] = 151,377	22,731,441	32.1	37.3
Ouellette-Kuntz H. et al. (2015) [40]	Any	50–64	[Intellectual] = 15,791	791,792	[Colon] = 32.0 [FOBT] = 18.3	47.2 26.4
Ramirez A. et al. (2005) [32]	Any	≥50	[Any disability] = 2,745,931	21,101,484	[Colon] = 41.9 [FOBT] = 22.5	43.4 23.1
Saito T. et al. (2024) [42]	FOBT/FIT	40–74	[Functional] = 85	5945	47.5	28.2
Shin D. W. et al. (2020) [37]	FOBT/FIT	≥50	[Functional] = 79,616 [Physical] = 1,027,532 [Hearing impairment] = 180,313 [Visual impairment] = 161,825 [Intellectual] = 33,953 [Learning] = 10,134 [Psychosocial] = 50,729	13,202,307	24.4 34.0 31.6 33.0 23.9 27.4 28.4	33.8
Steele C. B. et al. (2017) [33]	Any	50–75	[Physical] = 1136 [Hearing impairment] = 320 [Visual impairment] = 175 [Intellectual] = 216	4726	63.1 64.6 48.6 56.2	57.0
Yang S. et al. (2021) [34]	Colon/Sigma	50–75	[Intellectual] = 4868	19,914	59.9	58.9
Yarborough B. J. H. et al. (2018) [35]	FOBT/FIT	50–75	[Psychosocial] = 5007	87,438	42.2	38.3

**Table 4:** Summary of subgroup analyses by disability type and CRC screening type.

Subgroup	Screening Type	Studies Included	Pooled Estimate [OR (95%CI)]	Heterogeneity	
				I^2^	τ^2^
Functional disability	Any type	5 studies [25,27,37,38,41]	0.81 (0.53–1.23)	99.88%	0.22
	FOBT/FIT	3 studies [37,38,41]	0.59 (0.47–0.73)	99.64%	0.04
Physical disability	Any type	6 studies [26,29,33,37,38,41]	0.91 (0.71–1.16)	99.91%	0.09
	FOBT/FIT	3 studies [37,38,41]	0.77 (0.54–1.09)	99.96%	0.10
Hearing impairment	Any type	4 studies [33,37,38,41]	0.95 (0.76–1.19)	99.53%	0.05
	FOBT/FIT	3 studies [37,38,41]	0.87 (0.76–1.00)	98.96%	0.01
Visual impairment	Any type	6 studies [24,26,33,37,38,41]	0.74 (0.61–0.89)	98.88%	0.05
	FOBT/FIT	3 studies [37,38,41]	0.71 (0.50–1.00)	99.71%	0.09
Intellectual disability	Any type	7 studies [26,33,34,37,38,40,41]	0.65 (0.53–0.79)	99.37%	0.08
	FOBT/FIT	4 studies [37,38,40,41]	0.58 (0.49–0.69)	98.71%	0.03
Psychosocial disability	Any type	5 studies [23,30,37,39,41]	0.82 (0.69–0.97)	99.24%	0.04

## Data Availability

No new data were created or analyzed in this study. Data sharing is not applicable to this article.

## Supplementary Materials

The following supporting information can be downloaded at https://www.mdpi.com/article/10.3390/curroncol31110517/s1, Table S1: PRISMA 2020 checklist; Table S2: MEDLINE/PubMed search strategy; Table S3: Baseline demographic and socioeconomic characteristics; Table S4: Risk of bias; Figure S1: Pooled odds ratio estimates of any CRC screening participation by functional disability status; Figure S2: (a) Pooled odds ratio estimates of any CRC screening participation by physical disability status. (b) Pooled odds ratio estimates of FOBT or FIT participation by physical disability status; Figure S3: (a) Pooled odds ratio estimates of any CRC screening participation by hearing impairment status. (b) Pooled odds ratio estimates of FOBT or FIT participation by hearing impairment status; Figure S4: Pooled odds ratio estimates of FOBT or FIT participation by vision impairment status.
