# Supplementary material for: Disability and Participation in Colorectal Cancer Screening: A Systematic Review and Meta-Analysis
Source: Curr Oncol. 2024 Nov 10;31(11):7023–39. doi: 10.3390/curroncol31110517 (PMC11593103; doi:10.3390/curroncol31110517)
Supplement: Supplementary file 1 [file curroncol-31-00517-s001.zip › Supplement materials - Subgroup analyses by disability type and CRC screening type.pdf]

Subgroup analyses by disability type and CRC screening type

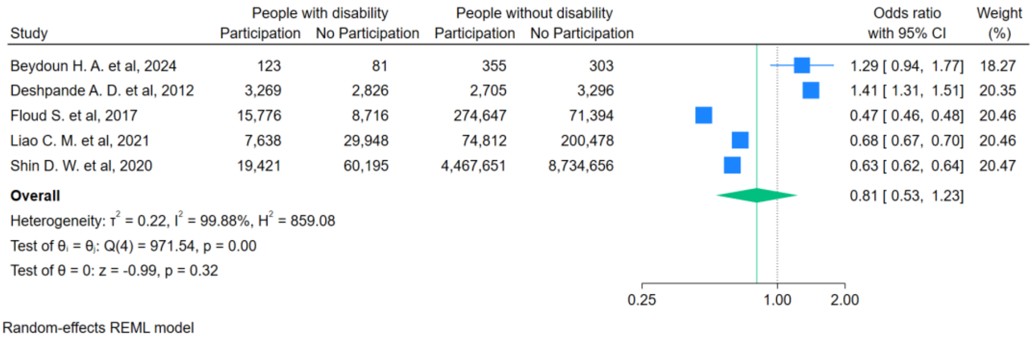

Figure S1. Pooled odds ratio estimates of any CRC screening participation by functional disability status.[25,27,37,38,41] Blue squares show the odds ratios (ORs) for individual studies, with horizontal lines for 95% confidence intervals and the green diamond indicates the overall pooled OR for all studies. The green dashed line indicates the overall pooled OR, allowing comparison with individual study ORs. The vertical dotted line indicates the null effect threshold.

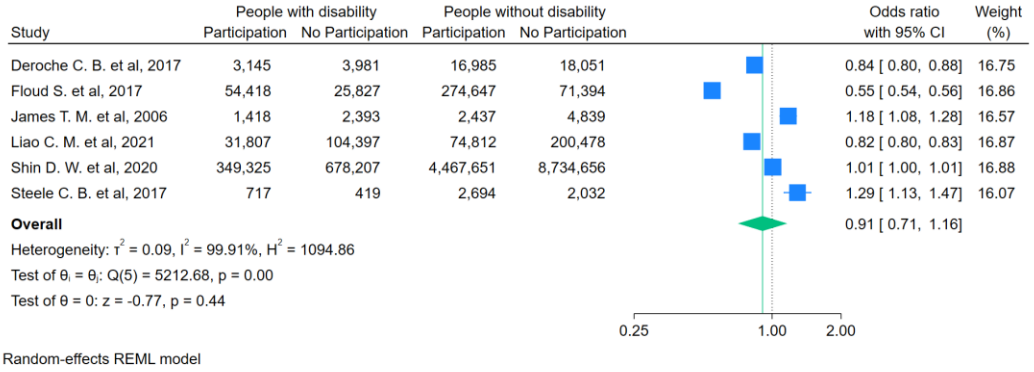

(**a**)

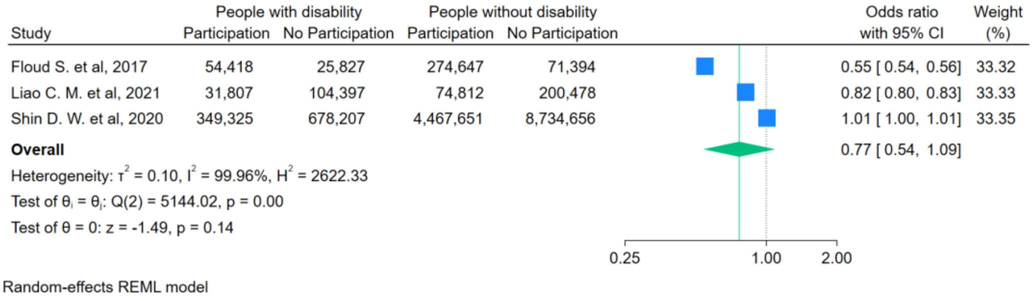

(**b**)

Figure S2. (**a**) Pooled odds ratio estimates of any CRC screening participation by physical disability status.[26,29,33,37,38,41] (**b**) Pooled odds ratio estimates of FOBT or FIT participation by physical disability status.[37,38,41] Blue squares show the odds ratios (ORs) for individual studies, with horizontal lines for 95% confidence intervals and the green diamond indicates the overall pooled OR for all studies. The green dashed line indicates the overall pooled OR, allowing comparison with individual study ORs. The vertical dotted line indicates the null effect threshold.

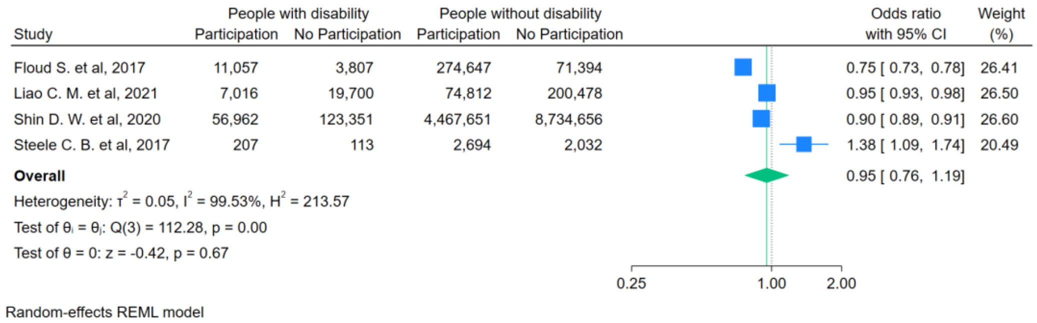

(**a**)

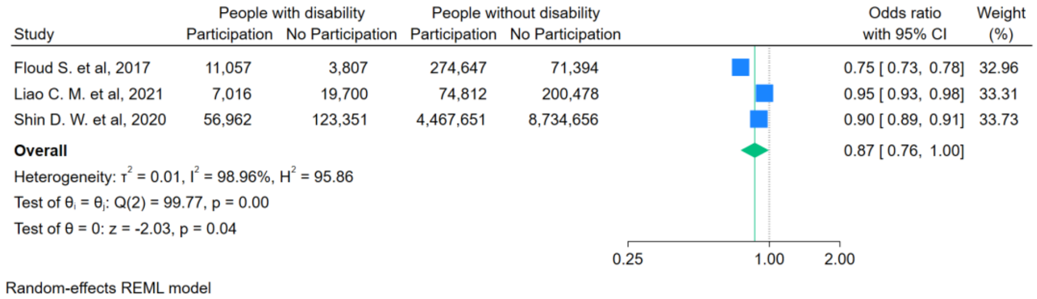

à

(**b**)

Figure S3b. (**a**) Pooled odds ratio estimates of any CRC screening participation by hearing impairment status.[33,37,38,41] (**b**) Pooled odds ratio estimates of FOBT or FIT participation by hearing impairment status.[37,38,41] Blue squares show the odds ratios (ORs) for individual studies, with horizontal lines for 95% confidence intervals and the green diamond indicates the overall pooled OR for all studies. The green dashed line indicates the overall pooled OR, allowing comparison with individual study ORs. The vertical dotted line indicates the null effect threshold.

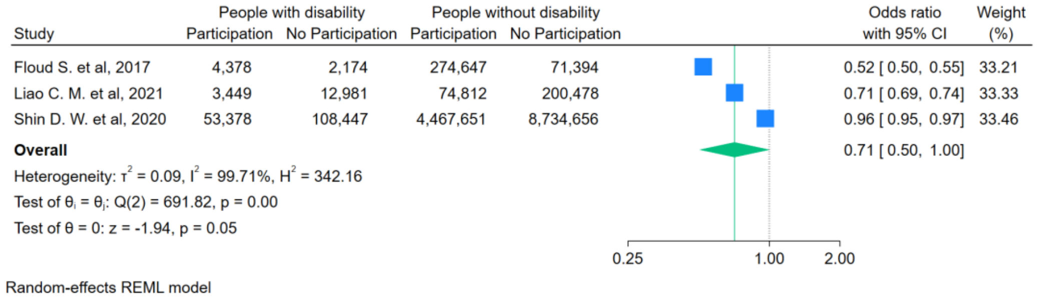

Figure S4. Pooled odds ratio estimates of FOBT or FIT participation by vision impairment status.[37,38,41] Blue squares show the odds ratios (ORs) for individual studies, with horizontal lines for 95% confidence intervals and the green diamond indicates the overall pooled OR for all studies. The green dashed line indicates the overall pooled OR, allowing comparison with individual study ORs. The vertical dotted line indicates the null effect threshold.
