# Supplementary material for: Disability and Participation in Colorectal Cancer Screening: A Systematic Review and Meta-Analysis
Source: Curr Oncol. 2024 Nov 10;31(11):7023–39. doi: 10.3390/curroncol31110517 (PMC11593103; doi:10.3390/curroncol31110517)
Supplement: Supplementary file 1 [file curroncol-31-00517-s001.zip › Supplement materials - Table S2. MEDLINE-PubMed search strategy.pdf]

Table S2. MEDLINE/PubMed search strategy.

Set	Key Words	Records retrieved
1	disabled person*	
2	disabilit*	
3	disabled people	
4	amputee*	
5	hearing impairment*	
6	hearing impaired person*	
7	hearing impaired people	
8	hearing loss	
9	vision impairment*	
10	visually impaired person*	
11	visually impaired people	
12	vision disorder*	
13	blindness	
14	mentally disabled person*	
15	mentally disabled people	
16	mentally ill person*	
17	mentally ill people	
18	mental disorder*	
19	mental retardation	
20	mental retard*	
21	psychiatric disabilit*	
22	mental health disabilit*	
23	mental health impairment*	
24	intellectual disabilit*	
25	cognitive impairment*	
26	developmental disabilit*	
27	mobility limitation*	
28	mobility impairment*	
29	physically disabled	
30	physical disabilit*	
31	dependent ambulation	
32	paraplegia	
33	quadriplegia	
34	activity limitation*	
35	functional limitation*	
36	communication limitation*	
37	participation limitation*	
38	self-help device*	
39	assistive technology*	
40	Set 1-39 were combined with 'OR'	942,884
41	mass screening	
42	screening	
43	screen	
44	screening program*	
45	program*	
46	cancer screening	

47	neoplasm screening	
48	detection	
49	early detection	
50	cancer detection	
51	cancer early detection	
52	colonoscopy	
53	sigmoidoscopy	
54	occult blood	
55	stool test	
56	Set 41-55 were combined with 'OR'	3,913,969
57	colorectal cancer*	
58	colorectal neoplasm*	
59	rectal cancer*	
60	rectal neoplasm*	
61	intestinal neoplasm*	
62	intestinal cancer*	
63	sigmoid neoplasm*	
64	sigmoid cancer*	
65	Set 57-64 were combined with 'OR'	242,397
66	Set 40, 56 and 65 were combined with 'AND'	280
67	Set 67 was limited to 23 May 2024	
