# Supplementary material for: Disability and Participation in Colorectal Cancer Screening: A Systematic Review and Meta-Analysis
Source: Curr Oncol. 2024 Nov 10;31(11):7023–39. doi: 10.3390/curroncol31110517 (PMC11593103; doi:10.3390/curroncol31110517)
Supplement: Supplementary file 1 [file curroncol-31-00517-s001.zip › Supplement materials - Table S3. Baseline demographic and socioeconomic characteristics.pdf]

Table S3. Baseline demographic and socioeconomic characteristics.

Author	Gender	Race	Income	Insurance	Marital status	Level of education
Allar B. G. et al (2023) [23]	Female = 54.3%<br>Male = 45.7%	White = 39.9%<br>Hispanic = 18.2%<br>Black = 17.5%<br>Other = 14.8%<br>Asian = 9.6%	$24,000-65,000 = 29.4%<br>$65,001-83,200 = 24.4%<br>$83,201-101,000 = 21.2%<br>$101,001-250,000 = 24.9%	Medicaid = 47.1%<br>Medicare = 26.9%<br>Commercial = 26.0%	Married/Single = 50.2%<br>Divorced = 31.4%<br>Widowed = 10.5%<br>Legally = 4.6%<br>Separated = 3.0%<br>Unknown = 0.3%	NA
Bennett K. J. et al (2016) [24]	Female = 51.0%<br>Male = 49.0%	White, non-Hispanic = 65.2%<br>Black, non-Hispanic = 11.9%<br>Hispanic = 15.5%<br>Other, non-Hispanic = 7.0%	<200% = 31.8%<br>200–400% = 30.8%<br>400% = 37.1%	Private insurance = 67.1%<br>Public insurance = 20.0%<br>Uninsured = 13.0%	NA	High school = 38.1%<br>High school diploma = 22.8%<br>Some college or more = 38.8%
Beydoun H. A. et al (2024) [25]	Female = 7.7%<br>Male = 92.3%	White,non-Hispanic = 76.9%<br>Black, non-Hispanic = 11.6%<br>Other, non-Hispanic = 5.4%<br>Hispanic = 5.9%	< $10,000 = 5%<br>$10,000-50,000 =73.9%<br>≥ $50,000 =21.0%	None = 0.9%<br>Any = 99.1%	Never married = 56.3%<br>Married = 12.2%<br>Widowed = 29.5%<br>Separated/divorced = 11.0%	No high-school diploma or GED = 4.3%<br>High-school graduate/high-school diploma = 41.4%<br>Some college or associate degree = 39.3%<br>Bachelor's degree = 9.5%<br>Master's degree or above = 5.4%
Deroche C. B. et al (2017) [26]	Female = 51.0%<br>Male = 49%	NA	NA	Medicaid only = 78.9%<br>Medicare only =16.5%<br>Both sources = 4.6%	NA	NA
Deshpande A.D. et al (2012) [27]	Female = 58.1%<br>Male = 41.9%	Non-Hispanic white = 79.6%<br>Non-Hispanic black = 9.5%<br>Non-Hispanic other = 3.5%	NA	Private insurance = 69.6%<br>Public insurance = 22.8%<br>No insurance = 7.6%	Married/partnered = 65.6%<br>Not married/partnered = 34.5%<br>Missing = 0.3%	Some high school or less = 18.4%<br>High school graduate = 32.2%<br>Some college = 24.9%

Author	Gender	Race	Income	Insurance	Marital status	Level of education
		Hispanic = 7.5%		Missing (n) = 0.1%		College graduate = 24.7%<br>Missing = 0.9%
Floud S. et al (2017) [38]	Female = 100%	White = 83.3%	NA	NA	Not married or living with a partner = 20.9%	No educational qualifications = 33.9%
Iezzoni L. I. et al (2016) [28]	NA	White = 84.6%<br>Black = 10.2%<br>Asian = 3.6%<br>Other, multiple races = 1.7%	Income less than poverty threshold: 8.7%	No health insurance: 10.6%	NA	Less than high school: 15.0%<br>High school: 26.9%<br>Some college/ associates degree: 25.8%<br>College and advanced degrees: 32.3%
James T. M. et al (2006) [29]	Female = 58.8%<br>Male = 41.2%	Non-Hispanic, White = 74.2%<br>Non-Hispanic, Black = 12.5%<br>Hispanic = 10.8%<br>Non-Hispanic, other = 2.5%	< $15,000 = 27.4%<br>$15,000–34,999 = 29.9%<br>$35,000–54,999 = 17.5%<br>≥ $55,000 = 25.2%	Yes = 92.5%<br>No = 7.5%	Married = 49.0%<br>Other = 51.0%	No high school diploma = 27.4%<br>High school diploma = 31.5%<br>Some college and beyond = 41.0%
Kim D. S. et al (2024) [36]	Famale = 54.5%<br>Male = 45.5%	NA	<50% = 42.6%<br>50-99% = 29.2%<br>100-149% = 14.8%<br>150-200% = 6.9%<br>≥200% = 6.6%	Medicaid = 3.5%<br>National health insurance, self-employed = 35.0%<br>National health insurance, employed = 61.5%	Unmarried, separated or divorced = 23.0%<br>Married = 77.0%	Elementary school graduate or less = 50.3%<br>Middle school graduate = 15.6%<br>High school graduate = 20.4%<br>College graduate or higher = 13.7%
Kirkøen B. et al (2023) [39]	Female = 52.7%<br>Male = 47.3%	NA	≤ NOK484,000 = 18.1%<br>NOK484,001–755,000 = 25.2%<br>NOK755,001–1,130,000 = 27.4%<br>> NOK1,130,000 = 29.2%	NA	Cohabit/married = 79.9%<br>Single/widow = 20.1%	Primary school = 16.5%<br>High school = 46.8%<br>1–4 years of university = 25.9%<br>4 years of university = 10.3%
Liao C. M. et al (2021) [41]	Female = 41.8%<br>Male = 58.2%	NA	low income=5.5%<br>≤ 17,280 NT$= 5.8%	NA	NA	NA

Author	Gender	Race	Income	Insurance	Marital status	Level of education
			17,281-22,800 NT$=41.7%<br>22,801-28,800 NT$=18.8%<br>28,801-36,30 NT$=13.7%<br>≥ 36,301 NT$=14.6%<br>(monthly salary in NT$ New Taiwan dollar)			
May F. P. et al (2019) [30]	Female = 17.4%<br>Male = 82.6%	White=74.1%<br>Black=16.9%<br>Hispanic=4.6%<br>Asian=0.5%<br>American Indian/Alaskan Native=0.8%<br>Native Hawaiian/other Pacific Islander=0.7%<br>Multirace=1.0%<br>Unknown=1.5%	Highest SES quintile=20.5%<br>SES quintile 2=20.0%<br>SES quintile 3=19.5%<br>SES quintile 4=19.5%<br>Lowest SES quintile=20.6%	NA	NA	NA
Murphy K. A. et al (2021) [31]	Female = 52.8%<br>Male = 47.2%	NA	NA	NA	NA	NA
Ouellette-Kuntz H. et al (2015) [40]	Female = 50.5%<br>Male = 49.5%	NA	1 (lowest)=17.9%<br>2=19.3%<br>3=19.4%<br>4=20.6%<br>5 (highest)=21.6%<br>Missing=1.2%	NA	NA	NA
Ramirez A. et al (2005) [32]	Female = 51.4%	White=62.6%	<10k US$ Income=8.1%	Currently uninsured=12.5%<br>Public-based health	Married=55.1%	Non–high-school graduate=15.8%

Author	Gender	Race	Income	Insurance	Marital status	Level of education
	Male = 48.6%	Multiracial=3.7%<br>African American=5.6%<br>Asian American=10.8%<br>American Indian/Alaska Native=0.4%<br>Pacific Islander=0.3%<br>Other ethnicity=16.6%	10-15k US$ Income=7.9%<br>15-20k US$ Income=8.6%<br>20-30k US$ Income=12.2%<br>30-40k US$ Income=11.3%<br>40-50k US$ Income=8.8%<br>50-60k US$ Income=6.9%<br>60-100k US$ Income=20.4%<br>>100k US$ Income=15.8%	insurance=24.3%<br>Private-based health insurance=60.1%		High school graduate=26.3%<br>Some college/trade certification=18.9%<br>Associate Arts/Associate Science=8.2%<br>Bachelor of Arts/Associate Science=19.0%<br>Postgraduate education=11,7%
Saito T. et al (2024) [42]	Female = 51.4%<br>Male = 48.6%	NA	Wealthy=6.0%<br>Nor poor, not wealthy=38.8%<br>Poor=55.2%	Employee insurance=60.5%<br>National Health Insurance=36.9%<br>Other=2.6%	Married=79.2%<br>Single=10.2%<br>Divorced/widowed=10.6%	Vocational school/junior college/community (technical) college/university/post-graduate school = 43.8%<br>High school = 46.0%<br>Primary/junior school = 10.2%
Shin D. W. et al (2020) [37]	NA	NA	NA	NA	NA	NA
Steele C. B. et al (2017) [33]	NA	NA	NA	NA	NA	NA
Yang S. et al (2021) [34]	Female = 53.9%<br>Male = 46.1%	Non-Hispanic White=73.6%<br>Non-Hispanic Black=10.9%<br>Non-Hispanic Other=6.3%<br>Hispanic=9.2%	NA	Insured = 94.0%<br>Uninsured = 6.0%	Single=10.6%<br>Live with spouse=55.8%<br>Other=33.6%	High school or less=35.9%<br>More than high school=64.1%
Yarborough B. J. H. et al (2018) [35]	Female = 54.2%<br>Male = 45.8%	White = 94.0%	NA	NA	NA	NA
