# Supplementary material for: Disability and Participation in Colorectal Cancer Screening: A Systematic Review and Meta-Analysis
Source: Curr Oncol. 2024 Nov 10;31(11):7023–39. doi: 10.3390/curroncol31110517 (PMC11593103; doi:10.3390/curroncol31110517)
Supplement: Supplementary file 1 [file curroncol-31-00517-s001.zip › Supplement materials - Table S4. Risk of bias.pdf]

**Table S4.** Risk of bias.

| Authors | Risk |
|---|---|
| Allar B. G. et al (2023) [23] | − |
| Bennett K. J. et al (2016) [24] | − |
| Beydoun H. A. et al (2024) [25] | − |
| Deroche C. B. et al (2017) [26] | − |
| Deshpande A.D. et al (2012) [27] | − |
| Floud S. et al (2017) [38] | − |
| Iezzoni L. I. et al (2016) [28] | − |
| James T. M. et al (2006) [29] | − |
| Kim D. S. et al (2024) [36] | − |
| Kirkøen B. et al (2023) [39] | − |
| Liao C. M. et al (2021) [41] | − |
| May F. P. et al (2019) [30] | − |
| Murphy K. A. et al (2021) [31] | + |
| Ouellette-Kuntz H. et al (2015) [40] | − |
| Ramirez A. et al (2005) [32] | − |
| Saito T. et al (2024) [42] | + |
| Shin D. W. et al (2020) [37] | − |
| Steele C. B. et al (2017) [33] | − |
| Yang S. et al (2021) [34] | − |
| Yarborough B. J. H. (2018) [35] | + |

− Low risk of bias; + High risk of bias
